# Supplementary material for: Interface controlled thermal resistances of ultra-thin chalcogenide-based phase change memory devices
Source: Nat Commun. 2021 Feb 3;12:774. doi: 10.1038/s41467-020-20661-8 (PMC7858634; doi:10.1038/s41467-020-20661-8)
Supplement: Supplementary file 1 — Supplementary Information [file 41467_2020_20661_MOESM1_ESM.pdf]

Interface controlled thermal resistances of ultra-thin chalcogenide-based phase change memory devices

Kiumars Aryana[1], John T. Gaskins[1], Joyeeta Nag[2], Derek A. Stewart[2], Zhaoqiang Bai[2], Saikat Mukhopadhyay[3], John C. Read[2], David H. Olson[1], Eric R. Hoglund[4], James M. Howe[4], Ashutosh Giri[5], Michael K. Grobis[2], and Patrick E. Hopkins*[1,4,6]

[1]Department of Mechanical and Aerospace Engineering, University of Virginia, Charlottesville, Virginia 22904, USA

[2]Western Digital Corporation, San Jose, CA 95119, USA

[3]NRC Research Associate at Naval Research Laboratory, Washington, DC 20375, USA

[4]Department of Materials Science and Engineering, University of Virginia, Charlottesville, Virginia 22904, USA

[5]Department of Mechanical, Industrial and Systems Engineering, University of Rhode Island, Kingston, RI 02881, USA

[6]Department of Physics, University of Virginia, Charlottesville, Virginia 22904, USA

*Corresponding Author: phopkins@virginia.edu

Supplementary Note 1 - Electron vs. phonon contribution in thermal conductivity

An important factor in the thermal transport mechanism in GST is the contribution from the electrons vs. phonons in the total thermal conductivity. Application of the Wiedmann-Franz (WF) law is a common approach that makes use of electrical resistivity for estimating the electronic contribution in thermal conductivity.

$$k = k_\mathrm{p} + k_\mathrm{e} \tag{1}$$

$$k_\mathrm{e} = LT/\rho \tag{2}$$

where k_p and k_e are thermal conductivities due to phonon and electron contribution, respectively, L is the Lorenz number, often assumed as the low temperature value of 2.44×10^{-8} W Ω K^{-2}, T is temperature, and ρ is the electrical resistivity. Lyeo and coworkers [1] reported negligible electronic contribution in *a*-GST and c-GST, while ∼70% contribution in h-GST based on electrical resistivity measurements. However, a survey of the data available in literature, as given in Supplementary Table 1 for the electrical resistivity of the h-GST reveals a significant variations among reported values for the electrical resistivity of h-GST, ranging by as much as an order of magnitude. This difference among the electrical resistivities could be partially due to the different deposition process, composition variation, annealing time, or different measurement techniques. For example, Bragaglia *et al.* [2] reported that the resistivity of the h-GST largely depends on the degree of order in vacancy layers. They showed that for single crystalline h-GST, where the vacancy layers are highly ordered, the electrical resistivity could be substantially lower than reported values.

According to these studies, in h-GST, depending on the degree of disorder the thermal conductivity can largely vary. This is consistent with the observation of a disorder-induced metal-insulator transition in h-GST [3]. However, as the system transitions towards more order, as well as increased electron thermal conductivity the lattice thermal conductivity is expected to increase. First principle calculations demonstrate that the lattice thermal conductivity of bulk h-GST can vary in the range of 0.87-1.67 W m^{-1} K^{-1} depending on the crystal orientation [4]. Similarly, using first principle calculations, Campi et al. [5] showed that by adding various scattering terms (Sb/Ge sublattice disorder and vacancies), the lattice thermal conductivity of bulk h-GST can be adjusted to reduce from an ideal value of ∼1.6 W m^{-1} K^{-1} to experimentally reported value of ∼0.45 W m^{-1} K^{-1}. A more focused study on the effect of MIT on thermal conductivity can be found in Ref. [6].

Supplementary Table 1. Electrical resistivity for GST measured by different groups [1–3, 7–9] and the corresponding electronic contribution to thermal conductivity calculated from WF.

-	Resistivity h-GST (mΩ cm)	Measurement Temperature (°C)	Annealed Temperature (°C)	$Ge_2Sb_2Te_5$ Thermal Conductivity (W m^{-1} K^{-1})
Kato and Tanaka [7]	∼3	100	580	0.2440
Nirschl et al. [8]	∼2	100	350	0.3660
Lee et al. [9]	∼0.84	25	300	0.87
Siegrist et al. [3]	∼0.8	100	300	0.9150
Lyeo et al. [1]	∼0.58	25	400	1.2513
Bragaglia et al. [2]	∼0.32	0	270	2.2875

Supplementary Note 2 - Thermal boundary conductance measurements

The thermal properties of $Ge_2Sb_2Te_4$ (GST) are measured via Time-domain Thermoreflectance (TDTR). The experimental procedure and the data analysis for this technique are extensively discussed elsewhere [10–12]. Supplementary Figure 1(a) shows the experimental data with its corresponding theoretical fit for 20 and 160 nm h-GST. To find the GST thermal conductivity and the thermal boundary conductance (TBC) between GST and W ($G_{GST/W}$), we perform measurements on various thicknesses of GST. This is due to the fact that the sensitivity of our measurements to thermal conductivity and TBC varies with respect to thickness and, therefore, by measuring thermal conductance across various thicknesses we can distinguish the thermal conductivity from that of TBC. According to the sensitivity analysis in Supplementary Figure 1 (b,c), for 20 nm GST, the sensitivity of our measurements to TBC is highest whereas in the 160 nm thick GST, the sensitivity to TBC is negligible. Therefore, we obtain the intrinsic thermal conductivity of GST from 160 nm film where the influence from TBCs are minimum and obtain the TBC from the 40 and 20 nm films where the resistance from TBCs are comparable to that of the GST film.

For this, we measure the thermal conductance across Ru/W/GST/W/Si for a 20 and 40 nm thick GST, which includes the contribution of all resistances, i.e., layers and their corresponding interfaces. In TBC measurements, although it makes more sense to use the thinnest GST films like 5 and 10 nm where the resistance due to the GST film is less, we refrained from taking these ultra-thin thicknesses into account since the thermal transport are not fully within a diffusive regime. For diffusive thermal transport, the mean free paths of heat carriers must be shorter than the thickness of the film [13]. As discussed in the main manuscript, the carriers mean free paths for W layer can be up to 10 nm.

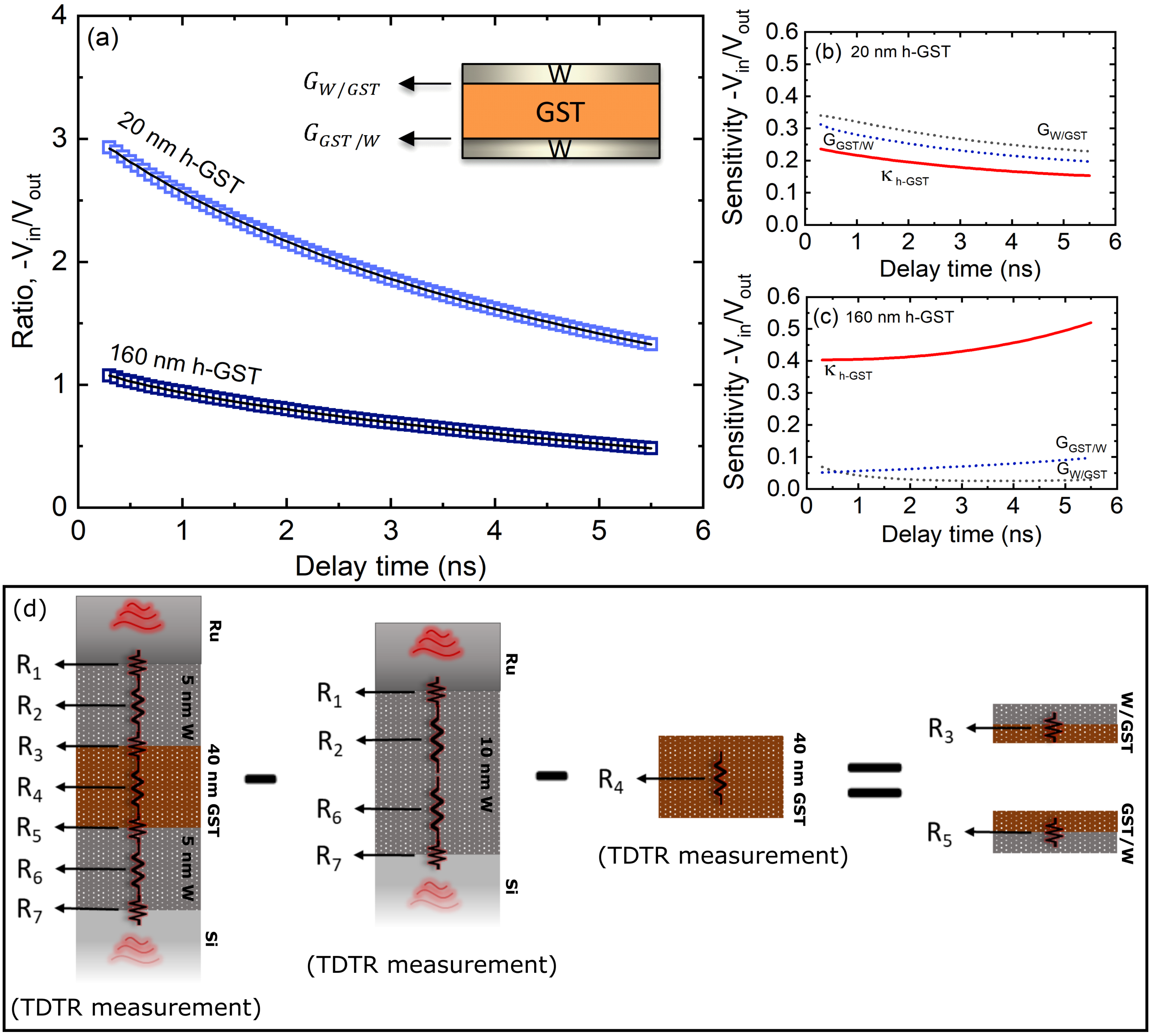

Supplementary Figure 1. Thermal model analyses.(a) Theoretical fit for 20 and 160 nm GST thickness, (b,c) Sensitivity to thermal conductivity and TBC on either side of GST layer for a 20 and 160 nm GST (d) Schematic representing the approach used in this study to find the thermal boundary conductance between GST and W.

In 20 and 40 nm thick GST, the existence of a thinner GST layer increases the sensitivity of our measurements to $G_{GST/W}$ (Supplementary Figure 1(b)). To find $G_{GST/W}$, as depicted in Supplementary Figure 1(d), we need to subtract the effect of all other resistors in the series from the total resistance. For this, since the 20 and 40 nm GST film yield a relatively small resistance between the Ru and Si, we can treat the entire stack (W/GST/W) as an interface and using a two layer model, measure the thermal conductance across the Ru/Si interface. The penetration depth in our measurement is on the order of ∼100 nm. The substrate is silicon which acts as a heat sink and therefore the resistance due to this layer is negligible. This leaves us with seven resistors between Ru and Si as depicted in the first schematic in Supplementary Figure 1(d).

Now, in order to deconvolve the thermal conductivity from that of the TBC, we need to know the intrinsic thermal conductivity of each layer as well as their corresponding TBCs. For this, using a different set of samples, we measure the thermal conductance across Ru/10 nm W/Si to account for the intrinsic thermal conductivity of W, Ru/W, and W/Si interfaces. Next, assuming the intrinsic thermal conductivity of 20 and 40 nm thick GST film is similar to that of the 160 nm, we subtract the resistance due to the intrinsic thermal conductivity of the 20 and 40 nm GST layer from the total resistance. In order to mathematically derive an equation for estimating the TBC between GST and W, we assume each layer and interface introduces a resistance to the thermal transport from the transducer to the substrate similar to the schematic in Supplementary Figure 1 (d). We can obtain the overall resistance of the stack between Ru and Si as follows:

$$\begin{aligned} R_{\text{total}} &= R_{\text{W}} + R_{\text{GST}} + R_{\text{W}} \\ &\quad + R_{\text{Ru/W}} + R_{\text{W/GST}} + R_{\text{GST/W}} + R_{\text{W/Si}}, \end{aligned} \tag{3}$$

Where R represents the thermal resistance and is defined as the inverse of thermal conductance, $R_{\text{total}} = 1/G_{\text{total}}$. Due to high thermal conductivity of W, the thermal resistance of the W layer compared to that of GST is negligible and can be dropped from Eq 3. Additionally, due to electronic transport of heat between the metals, the interfacial thermal resistance between metal-metal interface such as Ru/W is negligible. Furthermore, we assume the boundary conductance at the front and rear sides of the GST that are in contact with tungsten are identical ($R_{\text{W/GST}} = R_{\text{GST/W}}$). As a result, Eq. 3 can be simplified to:

$$R_{\text{total}} = R_{\text{GST}} + 2R_{\text{W/GST}} + R_{\text{W/Si}}, \tag{4}$$

In the above equation, except for the $R_{\text{W/GST}}$, other parameters can be measured from TDTR and TEM. Considering that the thermal resistance is the inverse of the thermal conductance, by rearranging the terms in Eq. 4 we can obtain an equation for GST/W thermal boundary conductance as follows:

$$\text{TBC}_{\text{GST/W}} = \frac{2}{(\frac{1}{G_{\text{total}}} - \frac{d_{film}}{k_{\text{160 nm GST}}} - \frac{1}{G_{\text{Ru/10 nm W/Si}}})}. \tag{5}$$

In this analysis, G_{total} is the total thermal conductance across the composite Ru/W/GST/W/Si stack and d_{film} is the thickness of the GST film. Assuming a similar top and bottom interface between GST and W, we multiply the obtained TBC by two (in the numerator) to estimate an individual

GST/W interface. Using Eq. 3, we provide an estimate for the $G_{W/GST}$ across different temperatures by averaging the values from 20 and 40 nm GST films. However, it must be noted that due to low thermal conductivity of the GST film in amorphous and cubic phases, the sensitivity to the $G_{W/GST}$ is not sufficient to enable us to directly fit for this parameter. Supplementary Figure 2 (a-c) shows the sensitivity of our measurements to parameters like thermal conductivity and TBC at each phases. There is negligible sensitivity to TBC in the amorphous phase. Upon transformation of a-GST to c-GST, the sensitivity to TBC increases but is still significantly lower than that of the thermal conductivity and therefore, would not affect the thermal conductivity measurements. The existence of low sensitivity to TBC in a-GST and c-GST, explains why the thermal conductivity for both 40 and 160 nm thick GST is similar up to 300 °C. However, as the c-GST transitions to h-GST, the sensitivity to the thermal conductivity decreases relative to those of the TBCs across the interfaces, and therefore, above 320 °C, the effective thermal conductivity is suppressed by the influence of interfaces.

Supplementary Figure 2 (d-f) shows our motivations behind using a lower bound in the main manuscript for $G_{W/GST}$ across different phases. These contour plots indicates the residual value for our model's fit relative to the best-fit with respect to the input thermal conductivity and $G_{W/GST}$. For this, a range of different values for the thermal conductivity and $G_{W/GST}$ are used to fit our model to the experimental data, and based on the amount of deviation from the best-fit value these plots are generated for different phases of GST. The blue region in these plots corresponds to the minimum deviation from the best-fit value. In other words, for any thermal conductivity and $G_{W/GST}$ that are taken from the blue region, the model generates the same quality of fit to the empirical data. This being said, since we can find the thermal conductivity of GST using the thick sample (160 nm), technically, we should be able to directly fit for the $G_{W/GST}$ in our thermal model using the 20 or 40 nm GST measurements. However, as demonstrated in Supplementary Figure 2 (d and e), due to lack of sensitivity in a-GST and c-GST, for any $G_{W/GST}$ that has a value higher than the red circle mark (20 and 110 MW m^{-2} K^{-1} for amorphous and cubic, respectively), the model produces a good fit. For example, in the amorphous phase, if we only fit for the thermal conductivity and fix $G_{W/GST}$ to any value between 20 MW m^{-2} K^{-1} and infinity, the model produces the same quality of fit to the experimental data. On the other hand, in h-GST (2 (f)), the $G_{W/GST}$ that are in the range of 45-115 MW m^{-2} K^{-1} can produce a good fit. This observation leads to prescribing a minimum limit to $G_{W/GST}$ for amorphous and cubic phases. In order to calculate a lower bound for the $G_{W/GST}$ across different phases, we assume 10% uncertainty in the measurement of parameters that play a role in the thermal transport such as thermal conductivity of GST and its thickness. The following table indicates the sign for calculation of uncertainty that leads to a lower bound for TBC shown

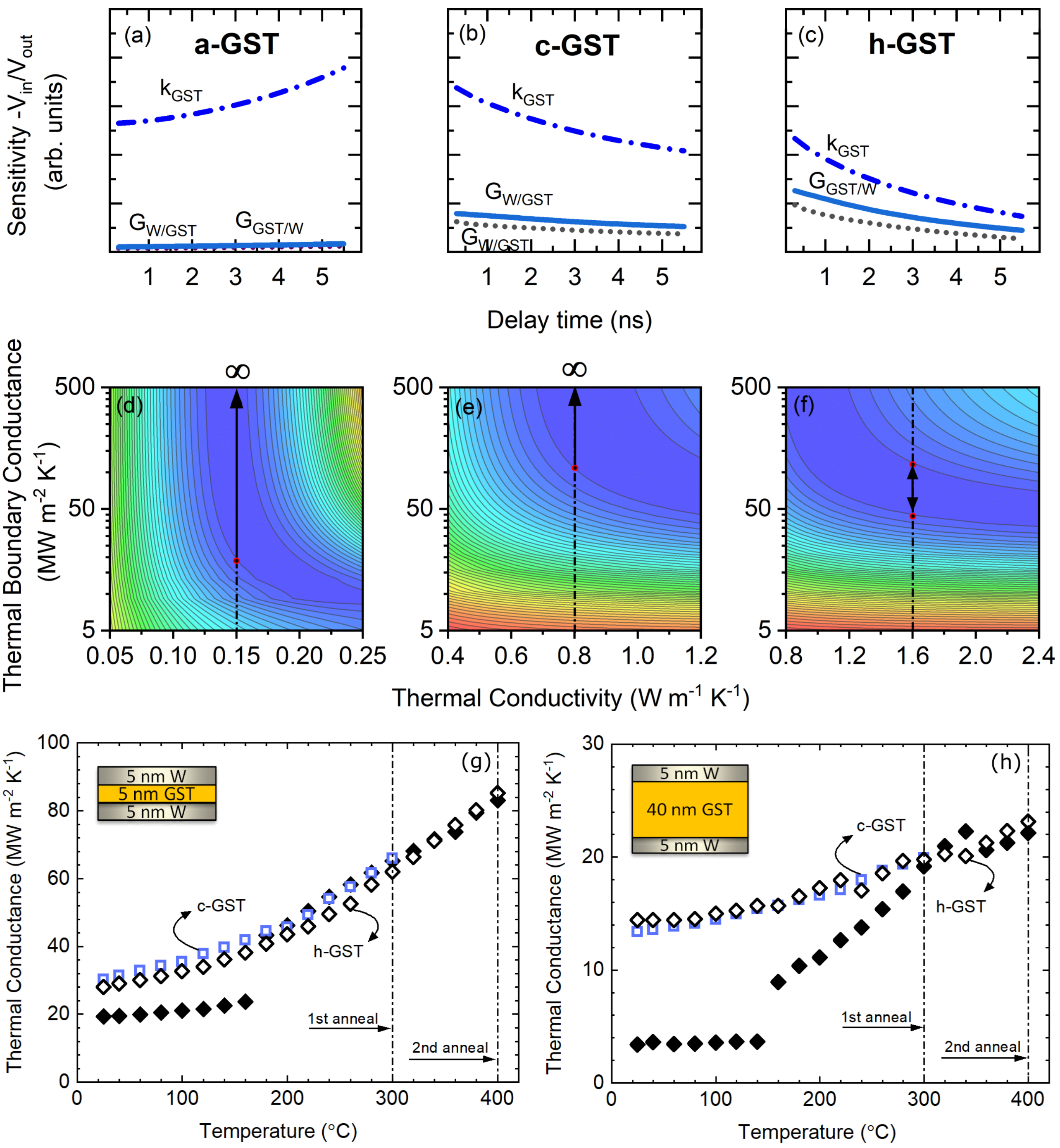

Supplementary Figure 2. Sensitivity and uncertainity analyses. (a-c) Sensitivity of thermal conductivity and top/bottom interfaces in a 40 nm thick GST as a function of delay time across different phases. (d-f) Residual contour of our model's fit relative to the best fit for $G_{GST/W}$ as a function of thermal conductivity in different phases of GST. The solid line arrows indicate the range where for any $G_{GST/W}$ input, the model produces a good fit with correct thermal conductivity. (g,h) Thermal conductance for 5 and 40 nm thick GST film, sandwiched between 5 nm W spacers.

in Supplementary Figure 3 (c,d) in the main manuscript. The sign is selected to ensure the lowest resulting $G_{W/GST}$.

Supplementary Table 2. The selected sign for setting a lower bound on TBC between GST and W given in the Fig 3 (c,d) in the main manuscript. G_{tot} is the thermal conductance across Ru/W/GST/W/Si

-	G_{tot}	$G_{Ru/10nmW/Si}$	d_{GST}	k_{GST}
Uncertainty	-10%	+10%	-10%	+10%

Similar to Supplementary Figure 3 (b) in the manuscript, in order to observe the thermal conductance of the stack when GST is in cubic vs.∼ hexagonal phase, we perform a similar measurement for 5 and 40 nm thick films. For this, we initially measure the thermal conductance for an as-deposited GST across different temperatures to determine the thermal conductance as the phase transition occurs. Then, we take another as-deposited sample, anneal it to 300 °C, then cool the sample down to room temperature, and measure its thermal properties at different temperatures. Again, the same sample was annealed to 400 °C and the thermal conductance was measured upon cooling. In this way, we obtained the thermal conductance across different temperatures for c-GST and h-GST. Supplementary Figure 2 (h,g) shows the results of these measurements. Annealing the 5 and 40 nm thick GST from 300 to 400 °C does not change the thermal conductance behaviour. This however, is surprising due to the fact that the room temperature thermal conductivity of c-GST and h-GST are ∼0.7 and ∼1.3 W m^{-1} K^{-1}, respectively. This provides additional evidence for the reduction of TBC in the hexagonal phase compared to cubic phase. We surmise that the reason we do not observe a pronounced difference between the c-GST and h-GST similar to Supplementary Figure 3(b) is because, in the case of 5 nm, the ballistic transport of carriers prevents us from observing the changes in the thermal boundary resistance (TBR), and for the case of 40 nm, we lose sensitivity to the TBC due to increased resistance of the GST film itself.

Supplementary Figure 3 (a,b) indicates the thermal conductance from room temperature to 400 °C for different thicknesses of GST sandwiched between 2 and 5 nm of W spacers, respectively. As can be seen, the thermal conductance is lower when the W thickness is 2 nm. This is also observed in the room temperature measurements as discussed in the main manuscript. Here, we observe a significant difference especially above the phase transition temperature ($\geq$ 150 °C). Above this temperature, the thermal conductance for 5 nm GST in both W thicknesses linearly increases with temperature. We attribute this to ballistic transport of phonon and electron across GST layer, as discussed in the main manuscript. It is worthwhile to mention that the effect of TBC between GST and W above 340 °C is most noticeable for GST thickness of 20 nm. This is because for thicknesses thinner than 20 nm, the

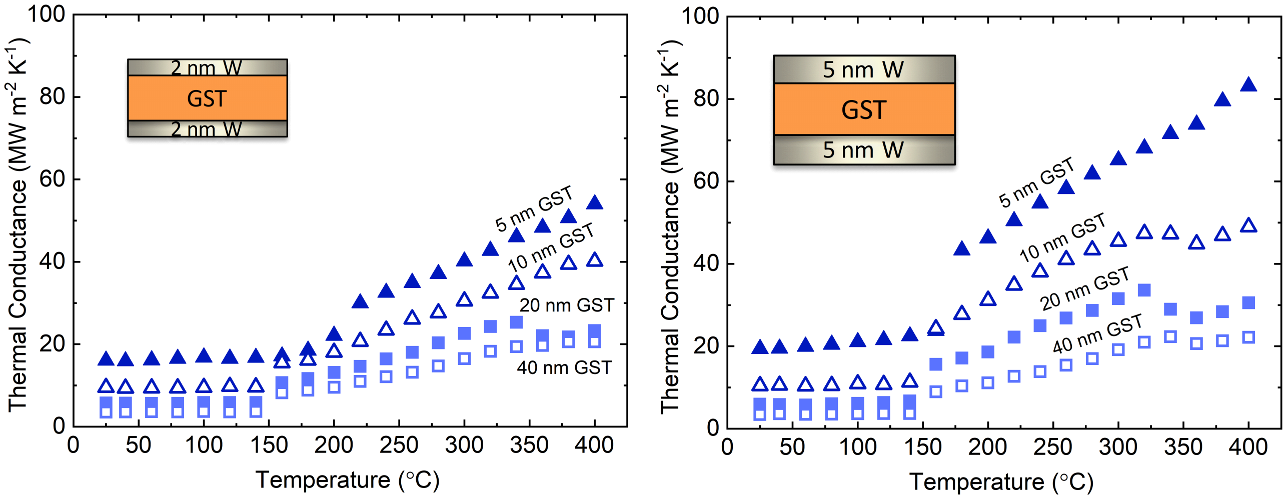

Supplementary Figure 3. Thermal conductance across Ru/W/GST/W/Si for different thicknesses of GST. (a) 5 nm and (b) 2 nm W spacers.

thermal transport is not fully diffusive. Whereas, for thicknesses larger than 20 nm, the GST layer resistance dominates the thermal transport. These plots clearly indicate that utilizing the 2 nm W is a better choice for electrode as it would result in a reduced thermal transport. We quantify this by coupling the experimental data with finite element simulations and find that merely reducing the W thickness can potentially lower the I_{reset} by ∼5%.

Supplementary Figure 4 (a) depicts the elemental mapping results for the GST layer determined from STEM, indicating the homogeneous distribution of Ge, Sb, and Te in a 5 nm GST layer. Supplementary Figure 4 (b) shows the TDTR measurement of the thermal conductivity as a function of time near the transition temperature (150 °C) for 40 nm GST. Prior to taking the measurements, the sample was heated up to 140 °C and after waiting long enough for the sample to equilibrate, the temperature was raised to 160 °C at a rate of 50 K/min. As can be seen, it takes nearly 2000 s for the GST layer to transition from amorphous to crystalline structure. Supplementary Figure 4 (c) indicates the thermal conductivity of thin GST films that have been annealed in a furnace for more than two hours at different temperatures. In our thin films, by applying a linear fit to the thermal resistance data as a function of the layer thickness, the thermal conductivity can be found. This method, however, only applies to GST for amorphous and cubic phase where the sensitivity to TBC is negligible, and we do not expect size effects in the thermal conductivity due to the relatively small mean free paths in the GST. As can be seen, the thermal conductivity increases as the annealing temperature increases and the values match the those obtained from the 160 nm measurements. On the other hand, in the hexagonal phase, since the reduction in TBC influences the measurements, the obtained resistance for the sample that is annealed to 400 °C is close or even higher than the 320 °C sample. Considering that h-GST has a factor of two higher thermal conductivity than c-GST, the measurement of higher

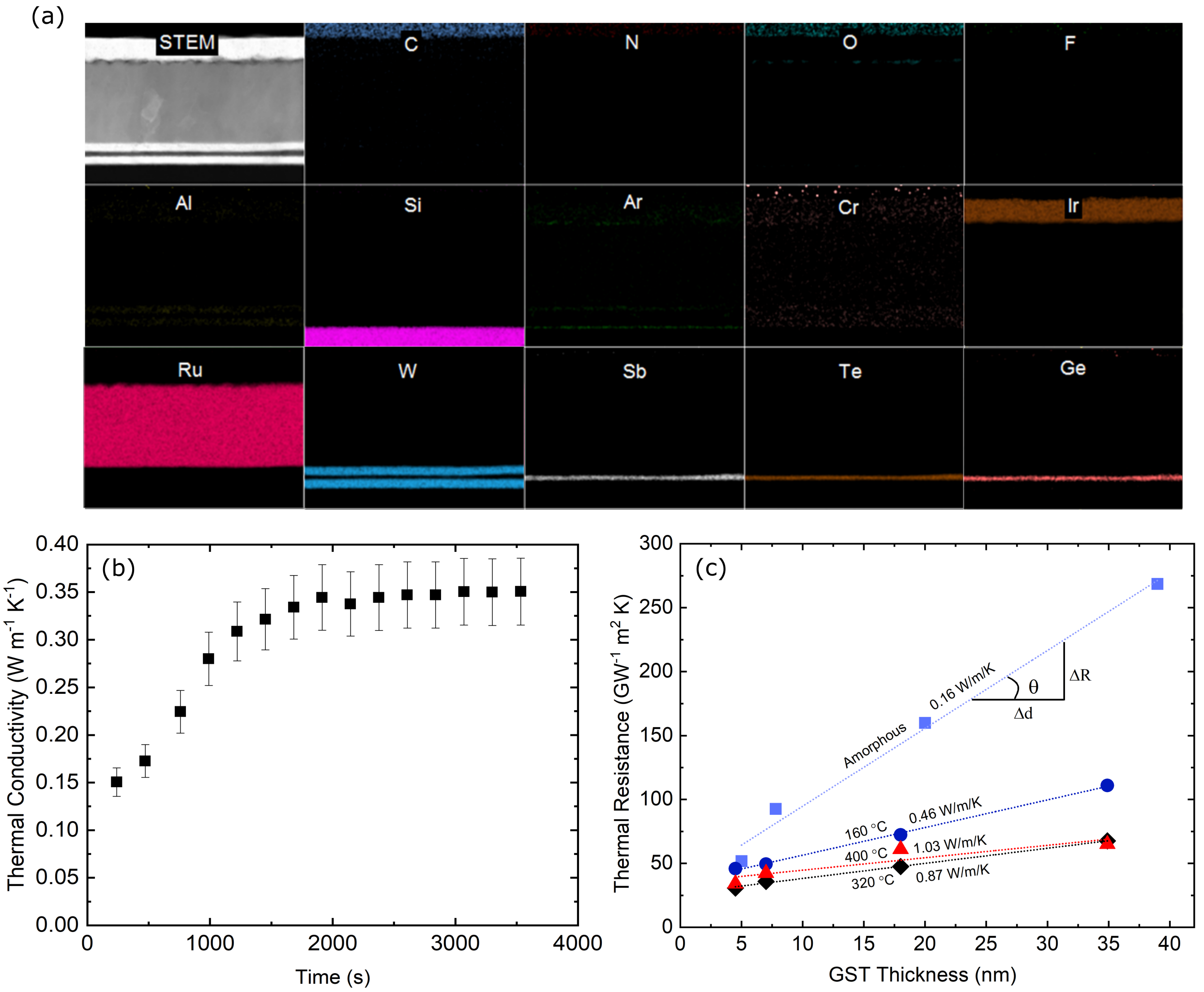

Supplementary Figure 4. (a) Elemental map for the constituents materials in the stack configuration studied (b) Thermal conductivity evolution as the GST transitions from amorphous to cubic crystalline at 140 °C to 160 °C as a function of time for a 40 nm thick GST film with W spacers. (c) Thermal conductivity of GST at different annealing temperatures. Note, the thermal conductivity of 400 °C annealed case is measured lower than reported in the manuscript due to the effect of reduced thermal boundary conductance.

thermal resistance for 400 °C annealed samples than 320 °C is unexpected. After performing *in situ* TEM and confirming that the GST film has not been damaged due to heating, we attributed this to the change in the thermal boundary conductance of GST with W layer.

Supplementary Note 3 - Why does interfacial resistance between GST and W change as the GST transitions from cubic to hexagonal?

As the GST undergoes the cubic to hexagonal phase transition, not only does the lattice structure change, but so do the electronic structure and the bonding. Obviously, the variation of several properties in GST makes it exceedingly difficult to pinpoint the exact reasons behind the observed reduction in TBC. Nonetheless, in order to provide more insight into the role of crystal structure and interfacial disorder on the observed transition in TBC, we conduct a series of molecular dynamics simulations of the TBC across cubic and hexagonal close packed interfaces of materials that have equivalent masses to W and GST using a 6-12 Lennard-Jones (LJ) potential. Lennard-Jones is a 2-body potential; therefore, the only free parameter is the distance between the atoms, and this enables us to create different lattice structures using the same potential. This therefore allows us to study the role of crystal structure and disorder on TBC without making any assumptions regarding changes in the bonding character from the cubic to hexagonal phases. To this extent, this highlights the advantages of conducting these molecular dynamics simulations using the LJ potential. Additionally, the simplicity of these potentials allows us to assess our hypotheses to general classes of materials, thus providing means to broadly study our posits of the origin of reduction in TBC across the crystalline phase transitions.

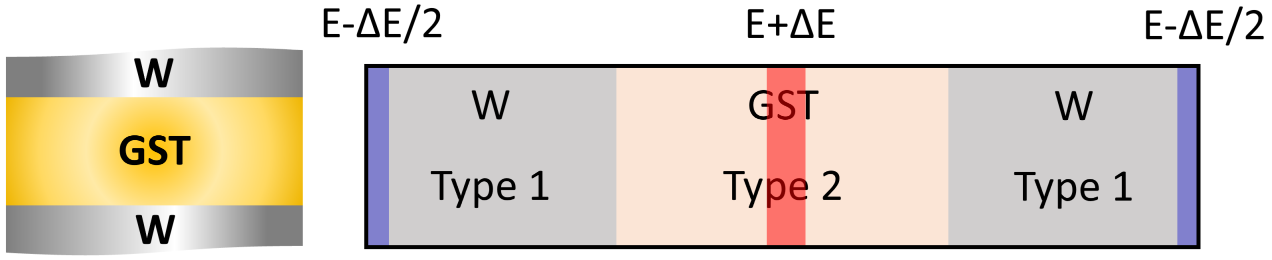

Supplementary Figure 5. Molecular dynamics simulations configuration. Simulation set up and the location of heat baths for the molecular dynamic calculations.

Since we are using LJ potentials to describe the crystalline W and GST films, in order to avoid confusion or misrepresentation, we call the section that represents W as *type 1* and the section that represents GST as *type 2*. With that in mind, we use parameters provided by Filippova et al. [14] for solid tungsten at room temperature (ϵ= 1.451420 eV and σ = 2.50374 nm). Although according to the paper, these parameters are supposed to result in a BCC lattice structure, we observe the lattice is unstable and tends to reorient to FCC structure. Nonetheless, we use this potential since our main purpose here is to investigate the effect of structural *changes* on TBC. For the atoms in type 2 (GST), we could use a LJ potential with softer bonding energy compared to that of tungsten, yet, to keep the

Supplementary Table 3. Thermal boundary conductance (TBC) and resistance (TBR) across the interface between different lattice structures. Disordered FCC/FCC and FCC/HCP interfaces are the cases where the initial phase of the atoms in type 2 were amorphous, however, during the course of simulation nucleated near the interfaces and transformed into a polycrystalline structure.

Interface	TBC (MW m^{-2} K^{-1})	TBR (GW^{-1} m^2 K)
ordered FCC/FCC	838	1.193
disordered FCC/FCC	1600	0.625
ordered FCC/HCP	1045	0.957
disordered FCC/HCP	2700	0.370

model as simple as possible, we use the same potential across all atom types in the GST and W. This allows us to only survey the effect of changes in the lattice structure. Thus, the only parameters that are different between type 1 (W) and type 2 (GST) are average atomic masses and number density. The atomic mass for type 1 is similar to that of W (183.84 u) and for type 2 is the arithmetic average of $Ge_2Sb_2Te_4$ (112.4 u). The number density for W and GST in our model is calculated to be $\sim 6.6 \times 10^{28}$ m^{-3} and $\sim 2.7 \times 10^{28}$ m^{-3} which stays relatively constant across all phases. For computational efficiency, a cutoff distance of 5.5 Å is used. For estimating the TBC at the interface between type 1 and type 2, we use a simulation box of 300 Å length with cross section area of 50×50 Å^2. In order to investigate the effect of disorder at the interface, we used a melt-quench technique to amorphize type 2 (GST) atoms. However, due to ordered interface of type 1, the amorphous structure nucleates near interface and turns into a thin FCC layer at the type-1/type-2 interface. We refer to this nucleated region as a disordered crystalline region which shows a higher TBC as compared to our "ordered" crystalline interfaces. The summary of our calculated TBC between different lattice structure are presented in supplementary table 3.

Our results suggest that a change in phase from cubic to HCP does not significantly change the thermal boundary conductance. However, structural disorder at the interface could play an important role in the reduction of TBC from the cubic to HCP phase in our measured data across the W/GST/W interfaces. This is consistent with previous computational and experimental observations regarding the effect of disorder at the interface on the enhancement of TBC [15–18]. Tian et al. [15] used a theoretical approach - atomistic Green's function- and showed that the interface roughness in Si/Ge can increase phonon transmission compared to an ideal sharp interface. They concluded that this effect is even more pronounced if the acoustic mismatch between the materials at the interface is large, which is the case for GST and W. Several molecular dynamics simulations [16, 19] have shown that compositionally disordered interfaces show higher TBCs than sharp interfaces. In addition, Gorham et al. [17] experimentally showed that TBC can increase across ion irradiated interfaces

of Al/native oxide/Si with sufficiently high ion dose due to compositional mixing and point defect formation. With respect to these previous works on the effect of disorder at the interface supported by our MD simulations, we hypothesize that one driving factor for the reduction in TBC from cubic to hexagonal phase could be due to the reduction of disorder rather than structural phase transition.

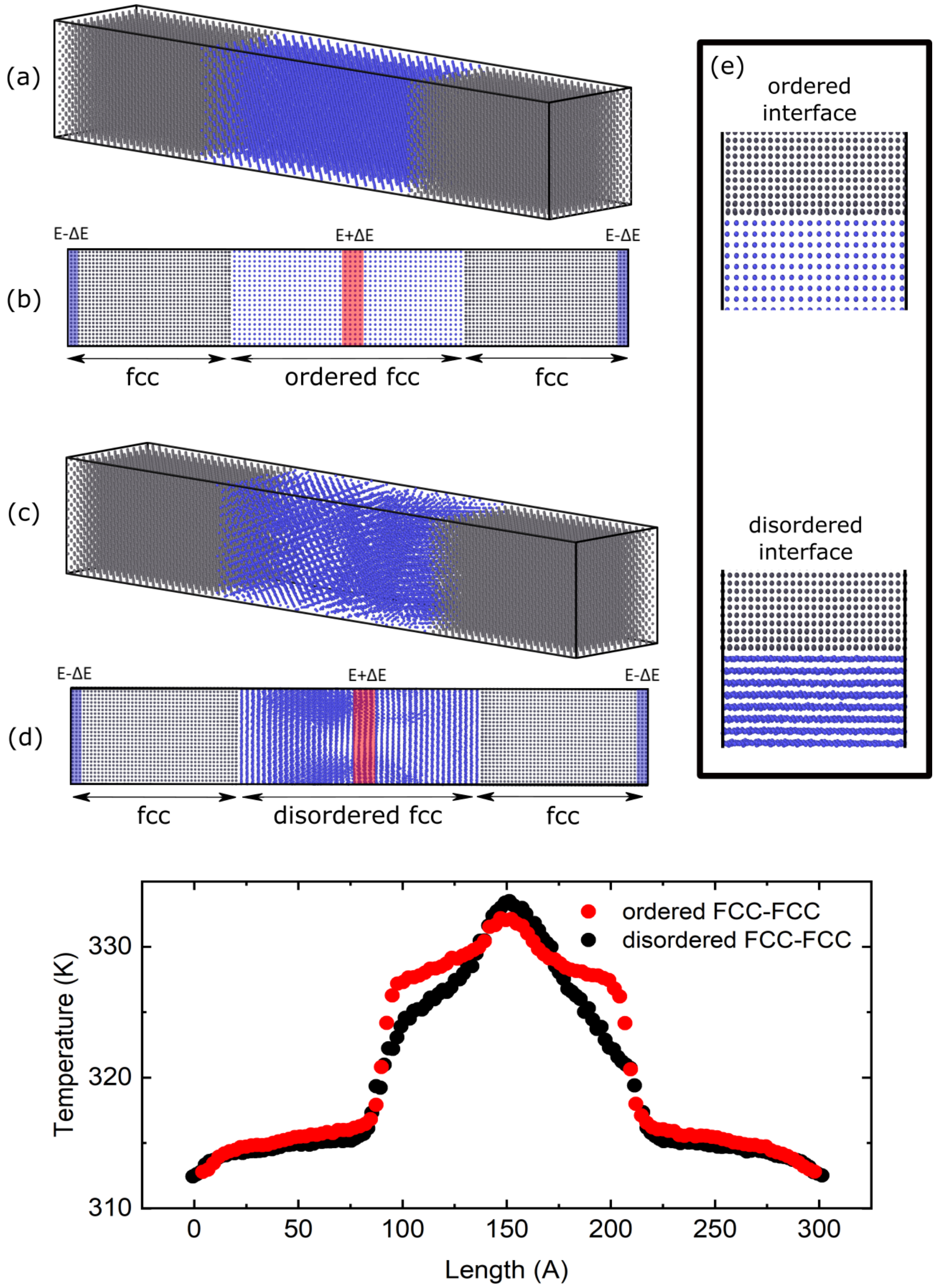

Supplementary Figure 6. Molecular dynamics simulation results for the system size of 50×50×300 Å^3. (a,b) 3D and 2D visualization of the atomic arrangement in the simulation after 6 ns for cubic/cubic/cubic structure. (c,d) 3D and 2D visualization of the atomic arrangement in the simulation after 6 ns for cubic/disordered cubic/cubic structure. The disordered cubic phase is the result of nucleation from an amorphous phase. (e) The quality of interface after 6 million timesteps for interfaces with different quality. (f) Temperature profile along the simulation box when ΔE = 1.5 eV/ps is added and subtracted from the hot and cold region depicted in red and blue (b,e). We calculate the TBC to be 838 MW m^{-2} K^{-1} and 1600 MW m^{-2} K^{-1} for ordered fcc/fcc and disordered fcc/fcc interfaces.

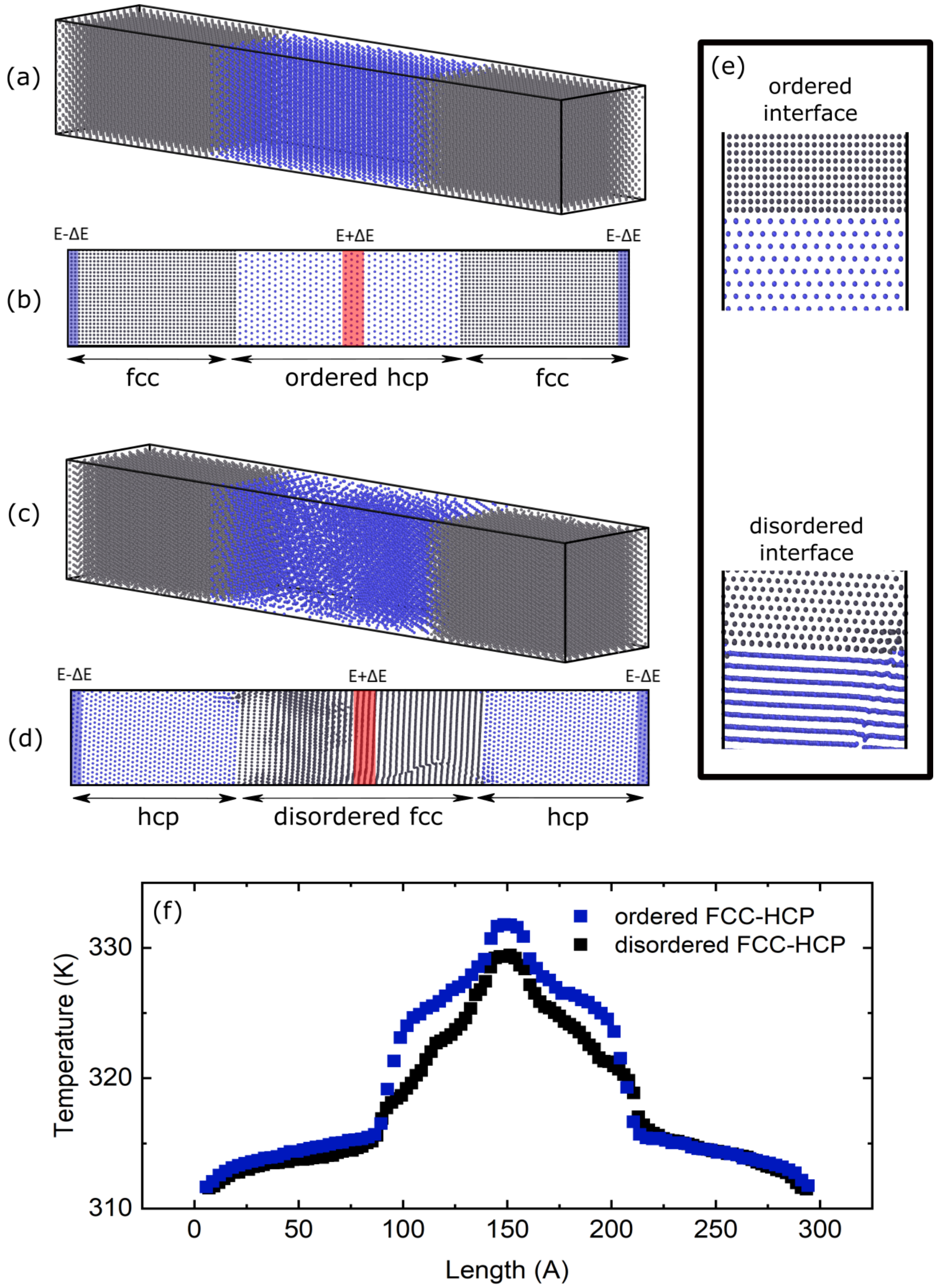

Supplementary Figure 7. Molecular dynamics simulation results for the system size of 50×50×300 Å^3. (a,b) 3D and 2D visualization of the atomic arrangement in the simulation after 6 ns for cubic/hexagonal/cubic structure. (c,d) 3D and 2D visualization of the atomic arrangement in the simulation after 6 ns for hexagonal/disordered fcc/hexagonal structure. The disordered cubic phase is the result of nucleation from an amorphous phase. (e) The quality of interface after 6 million timesteps for interfaces with different quality. (f) Temperature profile along the simulation box when $\Delta E = 1.5$ eV/ps is added and subtracted from the hot and cold region depicted in red and blue (b,e). We calculate the TBC to be 1045 MW m^{-2} K^{-1} and 2700 MW m^{-2} K^{-1} for ordered fcc/hcp and disordered fcc/hcp interfaces.

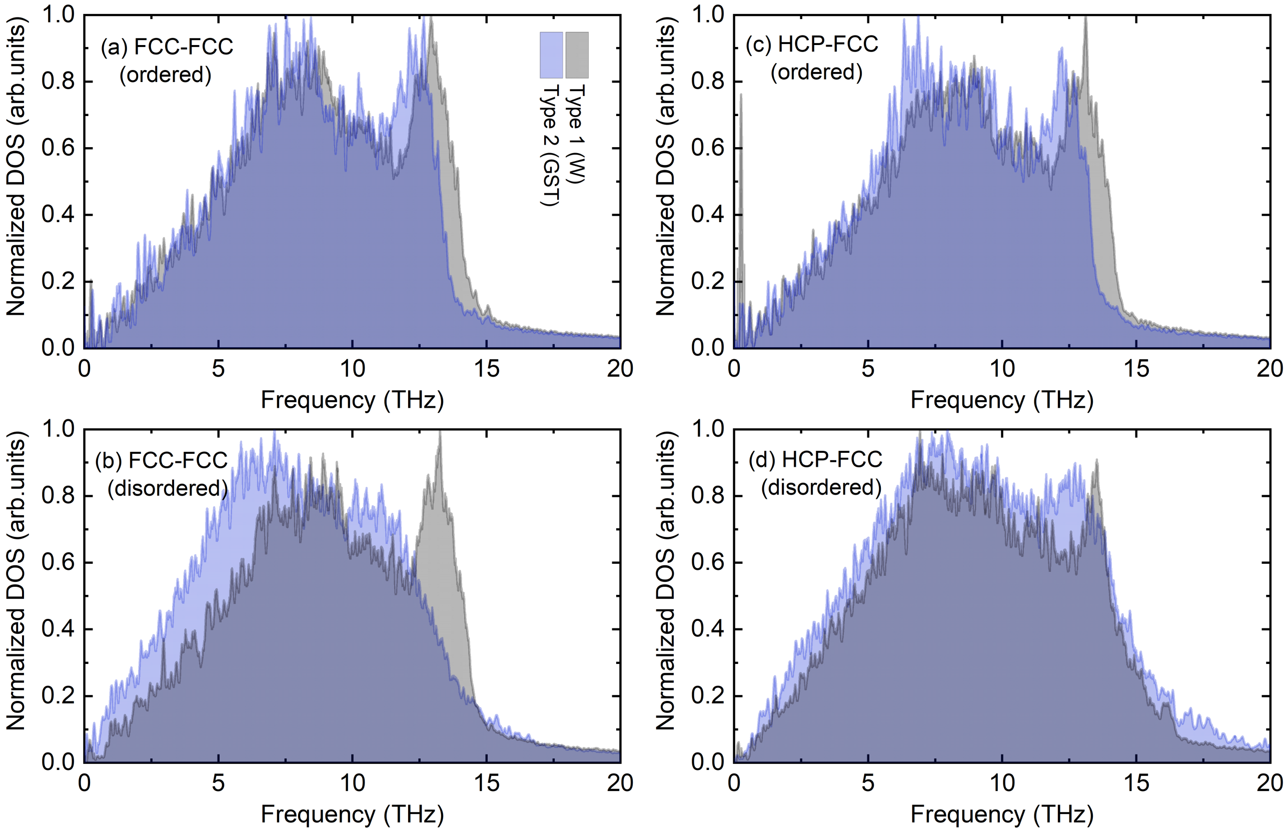

Supplementary Figure 8. Normalized density of states for type 1 and type 2 for different cases studied here. The Y-axis is dimensionless and has arbitrary units (arb. units).

Supplementary Note 4 - Sound speed measurement in ultra-thin GST

The phonon mean free path in materials plays an important role in the analysis of thermal conductivity which can be estimated with the knowledge of the sound speed, specific heat, and thermal conductivity [20]. Here, using the picosecond ultrasonic technique, we estimate the sound speed in GST for thicknesses less than 40 nm. In the configuration studied here, due to incorporation of multiple thin layers on top of each other (inset in Supplementary Figure 9 (b)), the interpretation of picosecond ultrasonic data can be complicated by the existence of reflections off different interfaces. Therefore, in order to decipher the picosecond ultrasonic results accurately, we began our measurements with a simple substrate/transducer sample and gradually added more layers to the stack to deconvolve the effects of additional layers on the picosecond ultrasonic signals. Supplementary Figure 9 (a) shows the time evolution of strain waves travelling through ruthenium transducers with varying thickness of tungsten interlayers on silicon substrate via picosecond ultrasonic measurements. As can be clearly seen in the Supplementary Figure 9 (a), every time a strain wave reflects off an interface and returns to the surface, a dip appears in the resultant thermoreflectivity decay curve which can be used to measure the exact thickness of each layer with knowledge of the sound speed in the material layers. The first decay curve corresponds to the base line where by measuring the time span between

the echoes, assuming a sound speed of 6150 m/s for ruthenium, we can calculate the distance travelled for each wave which corresponds to the thickness of the layers. With the addition of 10 and 20 nm of tungsten between the ruthenium and silicon, one would expect to observe a secondary dip in the decay curve owing to the incorporation of an additional interface. However, due to the relatively low acoustic mismatch at the Ru/W interface, most of the wave passes through the Ru/W interface with no significant reflection. In practice, the addition of tungsten layer manifests itself as an increase in the distance between the echoes in the the thermal decay curve. In other words, the interface between Ru and W does not have a noticeable impact on the transmission of strain waves and they are only reflected off of the W/Si interface. In the light of this result, we conclude the Ru/W interface will not affect the picosecond acoustic signal which simplifies the interpretation of data when a GST layer is added to the system.

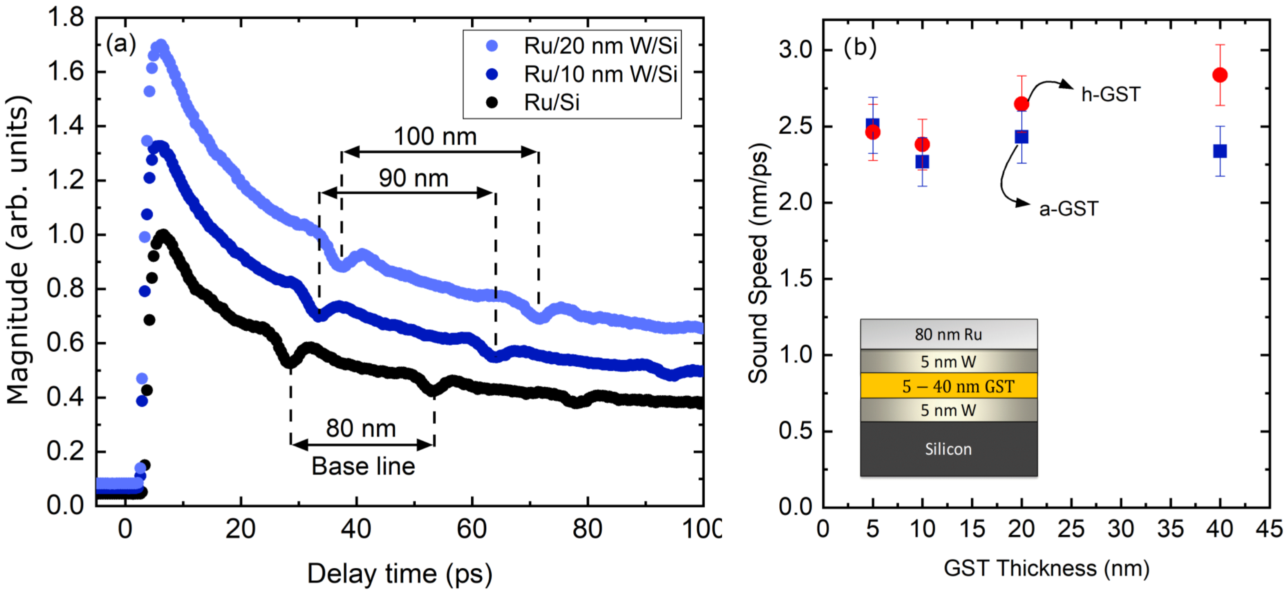

Supplementary Figure 9. Picosecond ultrasonic measurements (a) The TDTR signal for different stack configuration. (b) The measured sound speed for a-GST and h-GST at different thicknesses. The Y-axis is dimensionless and has arbitrary units (arb. units).

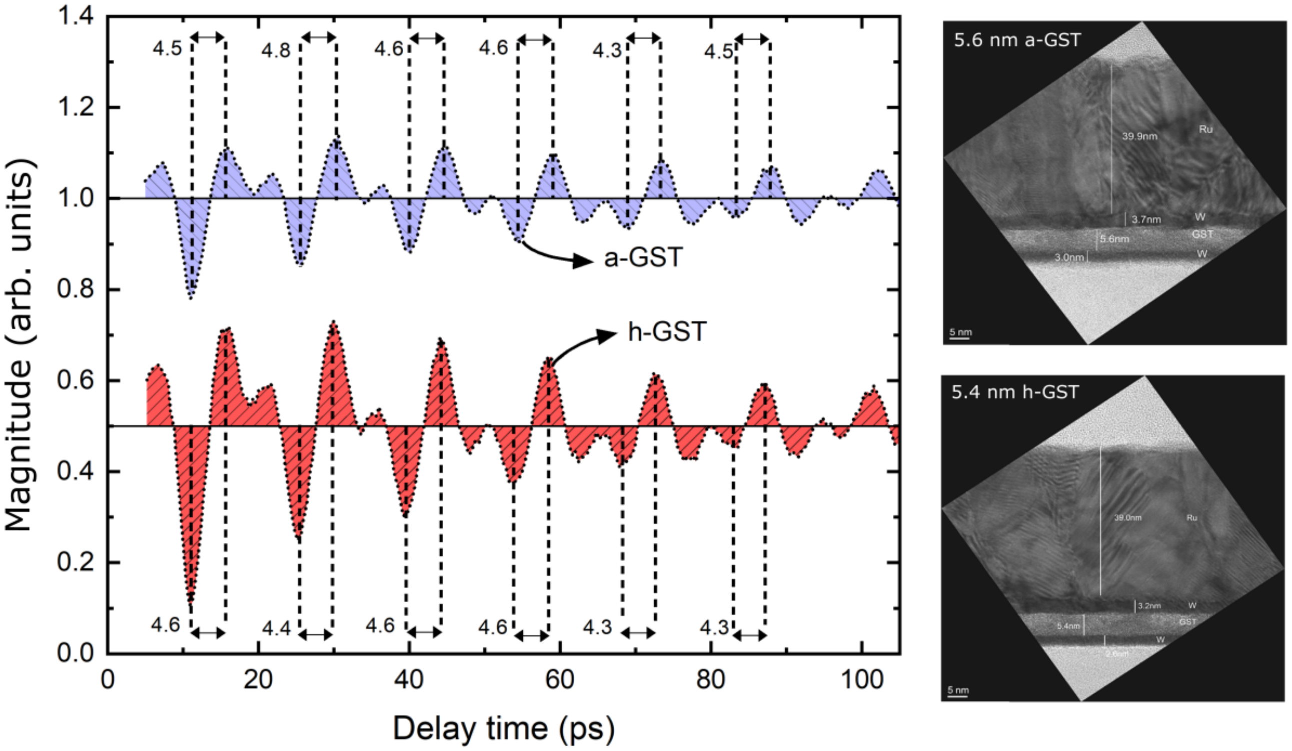

Supplementary Figure 10. Residual plot for picosecond ultrasonic measurement of a 5 nm thick GST in amorphous and hexagonal phase and the corresponding travelling time of strain waves in the GST layer. The Y-axis is dimensionless and has arbitrary units (arb. units).

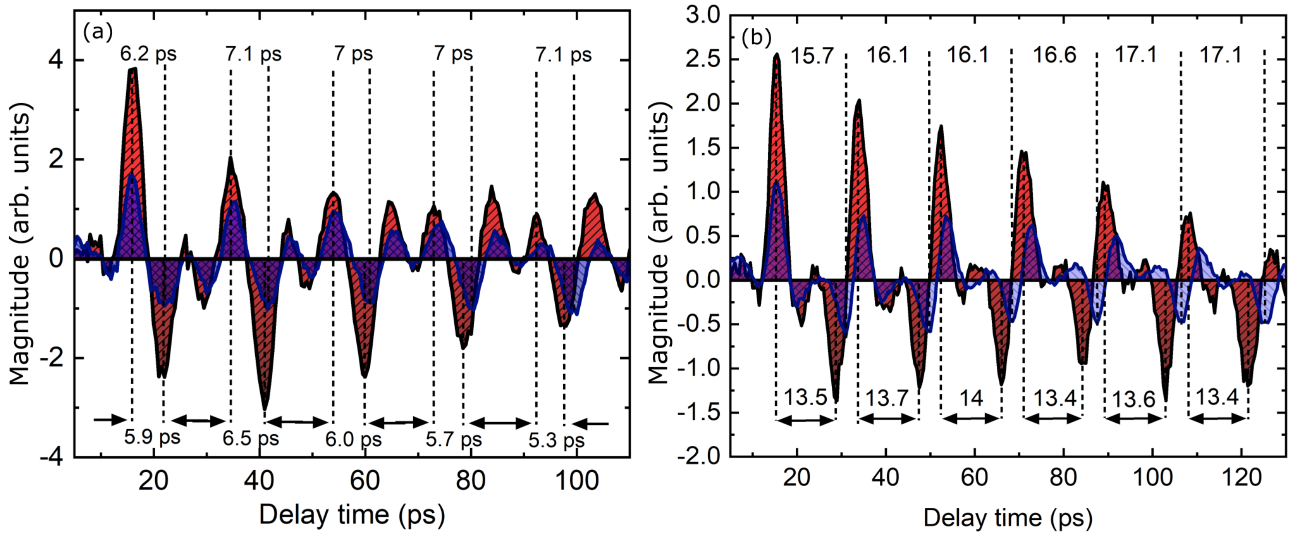

Supplementary Figure 11. Residual plot for picosecond ultrasonic measurement of (a) 10 nm and (b) 20 nm thick GST in amorphous and hexagonal phase and the corresponding travelling time of strain waves in the GST layer. The Y-axis is dimensionless and has arbitrary units (arb. units).

Supplementary Note 5 - Finite Element Simulation We model the propagation of strain waves using finite element (FE) simulations to ensure that our interpretation of the picosecond ultrasonic echoes are correct. In these simulations, the density, Poisson ratio, and the longitudinal sound speed are used as an input to determine the location of echoes in time. As such, the solid lines in Supplementary Figure 5 (a) in the main manuscript correspond to the simulation results and the dotted line corresponds to the picosecond residuals. As can be seen in Supplementary Figure 5 (a), the "humps" and "troughs" in the residual plots agree well with the simulations. The agreement for the location of the echoes between the simulations and the experiment confirms that they are not an artifact of measurement and are directly related to the reflection of the strain waves from the interfaces. The schematic in Supplementary Figure 5 (b) depicts the propagation of strain waves across different layers for the configuration studied here. As can be seen in Fig 5 (b) *i*, a strain wave is launched from the surface and travels across the Ru layer. Upon reaching the Ru/W interface, a lack of sufficient acoustic mismatch between Ru and W, allows the wave packet to completely pass through the interface without any interference (Fig 5 (b) *ii*). On the other hand, once the strain wave reaches the W/GST interface (Fig 5 (b) *iii*), as a result of large acoustic mismatch between W and GST, the wave is partially reflected and travels back to the surface and appears as upward "humps" in the residual plot. The other portion of the wave that passes the interface travels across the GST layer, and again, is partially reflected upon reaching the other GST/W interface where the consequence of this reflection appears as downward "troughs" in the residual plot. Using the time it takes for the strain waves to travel across the film, we can estimate the longitudinal sound speed.

Supplementary Figures 12 (a) and (b) depict the temperature profiles for a typical confined cell PCM device of 20 nm and 120 nm diameter, respectively, when the devices are subject to I_{reset} and reach thermal equilibrium. The I_{reset} is determined by ramping up the applied current until the 880 K isothermal lines (black lines in both Figures) touch the sidewalls of the PCM elements. The RESET condition is chosen in this way because 880 K is the melting temperature of $Ge_2Sb_2Te_5$ and we assume the portions of GST enclosed within the 880 K isothermal lines are molten and consequently amorphized after rapid cool-down. Therefore, the shunting paths are eliminated in such a condition, and the PCM devices are converted to the high resistance state. We repeat the same analysis for a mushroom cell geometry, Supplementary Figure 12 (c) and (d), and observe marginal change in the reset current compared to confined cell geometry.

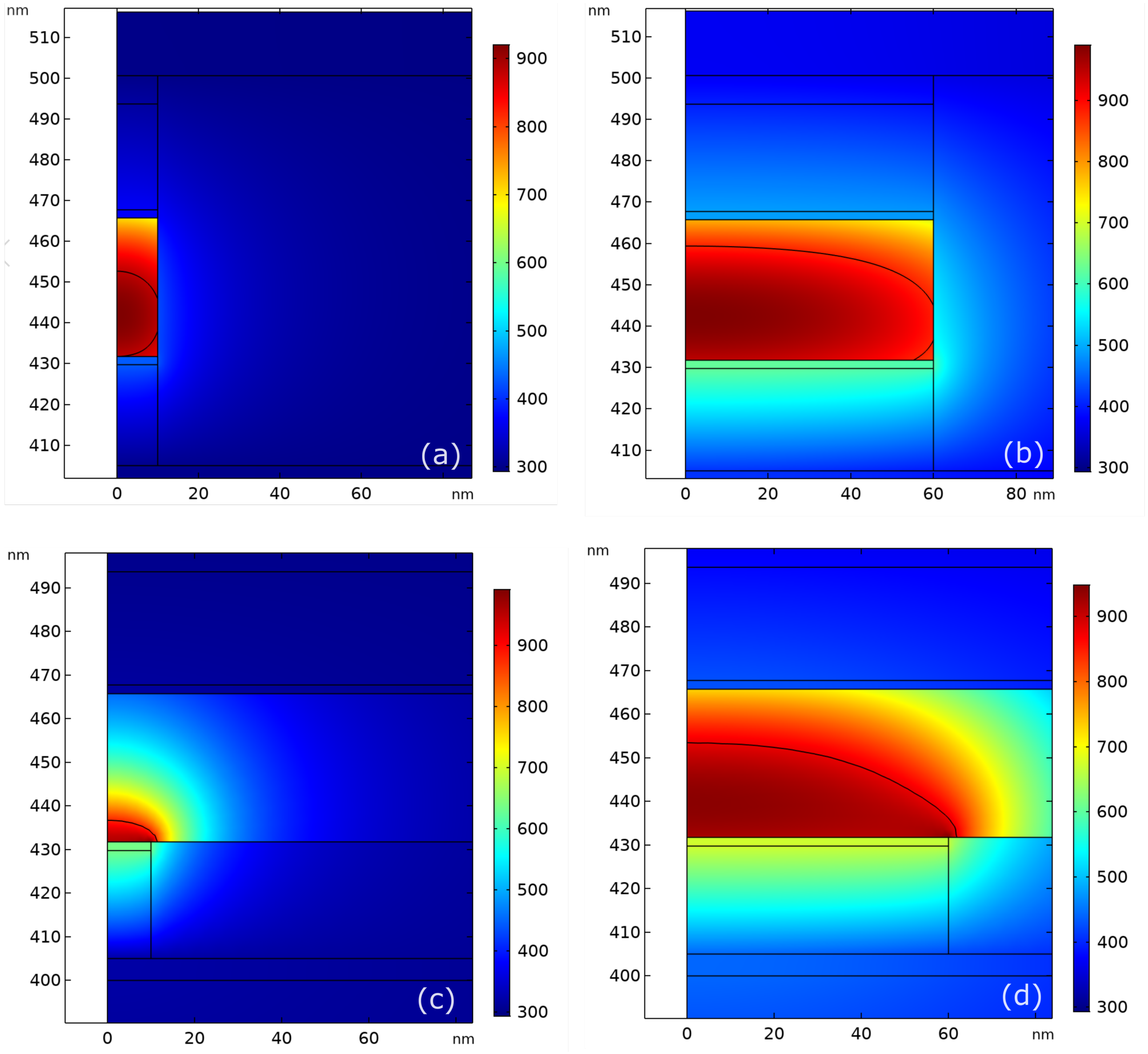

Supplementary Figure 12. Finite element simulations. (a,b) Temperature profile for a 35 nm thick GST memory cell with a confined cell geometry with 20 and 120 nm lateral size. (c,d) Temperature profile for a 35 nm thick GST memory cell with a mushroom cell geometry with 20 and 120 nm lateral size.

Supplementary Note 6 - Transmission Electron Microscopy In-situ heating was performed in a FEI Titan TEM at 300 kV equipped with a Gatan OneView camera. A Gatan heating holder (model 652) and Smart Set Hot Stage Controller (model 901) was used to heat the samples incrementally from room temperature by manually setting and ramping the applied current until the desired temperatures was attained. The manually ramping allowed for live continuous acquisition (in-situ movie) while transitioning from 25 to 240 °C and 240 to 400 °C, in addition to imaging and diffraction at 25, 240, and 400 °C where the temperature was held constant within ±5 °C. All diffraction patterns were taken using the OneView operating in DP mode to maximize the dynamic range of the camera. Selected-area diffraction patterns were acquired using an aperture collecting from an area 160 nm in diameter. The large collection region of the selected-area aperture relative to the thin-film thickness allowed sampling from both the GST layer and Si substrate providing a self-consistent calibration for GST amorphous ring patterns and crystalline diffraction patterns.

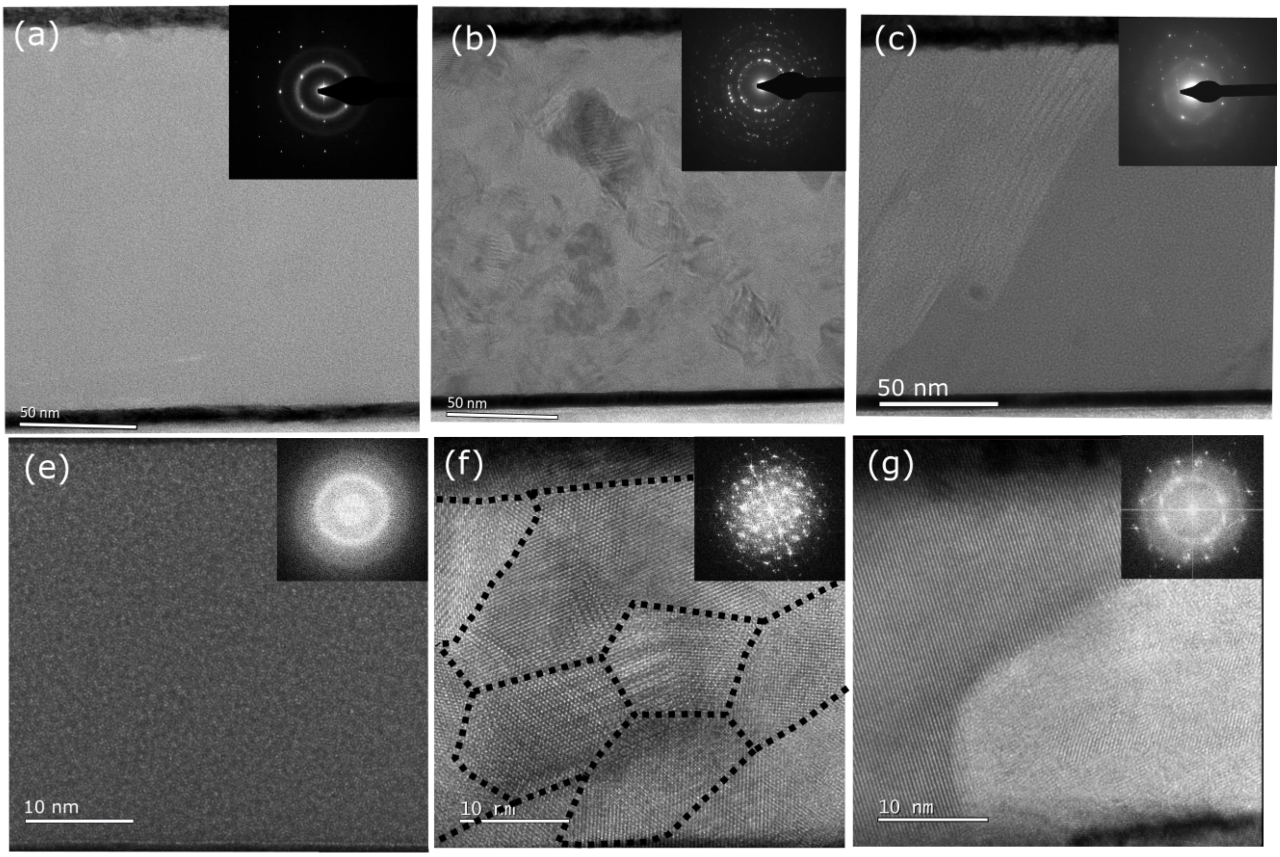

Supplementary Figure 13. Transmission electron microscopy for a-GST, c-GST, and h-GST phases and their corresponding diffraction patterns. (a-c) 160 nm GST, (e-g) 40 nm GST film.

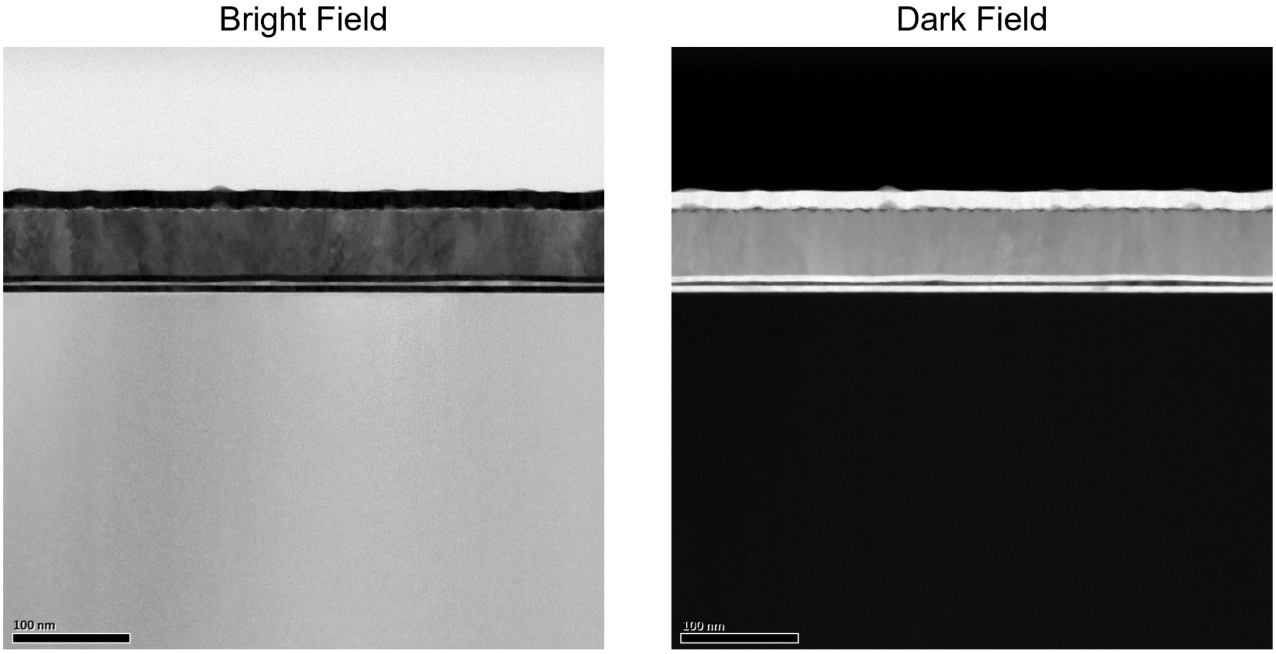

Supplementary Figure 14. Scanning transmission electron microscopy images for 5 nm thick a-GST.

References

[1] Ho-Ki Lyeo, David G Cahill, Bong-Sub Lee, John R Abelson, Min-Ho Kwon, Ki-Bum Kim, Stephen G Bishop, and Byung-ki Cheong. Thermal conductivity of phase-change material ge 2 sb 2 te 5. *Applied Physics Letters*, 89(15):151904, 2006.

[2] Valeria Bragaglia, Fabrizio Arciprete, Wei Zhang, Antonio Massimiliano Mio, Eugenio Zallo, Karthick Perumal, Alessandro Giussani, Stefano Cecchi, Jos Emiel Boschker, Henning Riechert, et al. Metal-insulator transition driven by vacancy ordering in gesbte phase change materials. *Scientific reports*, 6:23843, 2016.

[3] T Siegrist, P Jost, H Volker, M Woda, P Merkelbach, C Schlockermann, and M Wuttig. Disorder-induced localization in crystalline phase-change materials. *Nature materials*, 10(3):202–208, 2011.

[4] Saikat Mukhopadhyay, Lucas Lindsay, and David J Singh. Optic phonons and anisotropic thermal conductivity in hexagonal ge 2 sb 2 te 5. *Scientific reports*, 6:37076, 2016.

[5] Davide Campi, Lorenzo Paulatto, Giorgia Fugallo, Francesco Mauri, and Marco Bernasconi. First-principles calculation of lattice thermal conductivity in crystalline phase change materials: Gete, sb 2 te 3, and ge 2 sb 2 te 5. *Physical Review B*, 95(2):024311, 2017.

[6] KS Siegert, FRL Lange, ER Sittner, H Volker, C Schlockermann, T Siegrist, and M Wuttig. Impact of vacancy ordering on thermal transport in crystalline phase-change materials. *Reports on Progress in Physics*, 78(1):013001, 2014.

[7] Takayuki Kato and Keiji Tanaka. Electronic properties of amorphous and crystalline ge2sb2te5 films. *Japanese journal of applied physics*, 44(10R):7340, 2005.

[8] T Nirschl, JB Philipp, TD Happ, Geoffrey W Burr, Bipin Rajendran, M-H Lee, A Schrott, M Yang, M Breitwisch, C-F Chen, et al. Write strategies for 2 and 4-bit multi-level phase-change memory. In *2007 IEEE International Electron Devices Meeting*, pages 461–464. IEEE, 2007.

[9] Jaeho Lee, Elah Bozorg-Grayeli, SangBum Kim, Mehdi Asheghi, H-S Philip Wong, and Kenneth E Goodson. Phonon and electron transport through ge2sb2te5 films and interfaces bounded by metals. *Applied Physics Letters*, 102(19):191911, 2013.

[10] David G Cahill. Analysis of heat flow in layered structures for time-domain thermoreflectance. *Review of scientific instruments*, 75(12):5119–5122, 2004.

[11] Aaron J Schmidt, Xiaoyuan Chen, and Gang Chen. Pulse accumulation, radial heat conduction, and anisotropic thermal conductivity in pump-probe transient thermoreflectance. *Review of Scientific Instruments*, 79(11):114902, 2008.

[12] Patrick E Hopkins, Justin R Serrano, Leslie M Phinney, Sean P Kearney, Thomas W Grasser, and C Thomas Harris. Criteria for cross-plane dominated thermal transport in multilayer thin film systems during modulated laser heating. *Journal of Heat Transfer*, 132(8):081302, 2010.

[13] RB Wilson and David G Cahill. Anisotropic failure of fourier theory in time-domain thermoreflectance experiments. *Nature communications*, 5:5075, 2014.

[14] VP Filippova, SA Kunavin, and MS Pugachev. Calculation of the parameters of the lennard-jones potential for pairs of identical atoms based on the properties of solid substances. *Inorganic Materials: Applied Research*, 6(1):1–4, 2015.

[15] Zhiting Tian, Keivan Esfarjani, and Gang Chen. Enhancing phonon transmission across a si/ge interface by atomic roughness: First-principles study with the green's function method. *Physical Review B*, 86(23):235304, 2012.

[16] Timothy S English, John C Duda, Justin L Smoyer, Donald A Jordan, Pamela M Norris, and Leonid V Zhigilei. Enhancing and tuning phonon transport at vibrationally mismatched solid-solid interfaces. *Physical review B*, 85(3):035438, 2012.

[17] Caroline S Gorham, Khalid Hattar, Ramez Cheaito, John C Duda, John T Gaskins, Thomas E Beechem, Jon F Ihlefeld, Laura B Biedermann, Edward S Piekos, Douglas L Medlin, et al. Ion irradiation of the native oxide/silicon surface increases the thermal boundary conductance across aluminum/silicon interfaces. *Physical Review B*, 90(2):024301, 2014.

[18] Ashutosh Giri and Patrick E Hopkins. A review of experimental and computational advances in thermal boundary conductance and nanoscale thermal transport across solid interfaces. *Advanced Functional Materials*, 30(8):1903857, 2020.

[19] Ashutosh Giri, Patrick E Hopkins, James G Wessel, and John C Duda. Kapitza resistance and the thermal conductivity of amorphous superlattices. *Journal of Applied Physics*, 118(16):165303, 2015.

[20] J-L Battaglia, Andrzej Kusiak, Vincent Schick, Andrea Cappella, Claudia Wiemer, Massimo Longo, and Enrico Varesi. Thermal characterization of the si o 2-ge 2 sb 2 te 5 interface from room temperature up to 400 c. *Journal of Applied Physics*, 107(4):044314, 2010.
