# Supplementary material for: Interface controlled thermal resistances of ultra-thin chalcogenide-based phase change memory devices
Source: Nat Commun. 2021 Feb 3;12:774. doi: 10.1038/s41467-020-20661-8 (PMC7858634; doi:10.1038/s41467-020-20661-8)
Supplement: Supplementary file 4 — Description of Additional Supplementary Files [file 41467_2020_20661_MOESM4_ESM.pdf]

Description of Additional Supplementary Files

Supplementary Movie 1

Transmission electron microscopy with in situ heating, showing phase transition from amorphous to cubic (150 C) for 160 nm GST film.

Supplementary Movie 2

Transmission electron microscopy with in situ heating, showing phase transition from cubic to hexagonal (340 C) for 160 nm GST film.
